# Application of Doctor-Nurse-Patient Integration Management Mode in Patients with Endometriosis

**Published:** 2018-10

**Authors:** Yao CHEN, Zhihuai MO, Jianyong CAO, Zhaojuan SU, Yuexia ZHANG, Fang CHEN, Jianying WANG

**Affiliations:** 1. Dept. of Obstetrics, The Fifth Affiliated Hospital Sun Yat-Sen University, Zhuhai, China; 2. Dept. of Neurology, The Fifth Affiliated Hospital Sun Yat-Sen University, Zhuhai, China

**Keywords:** Doctor-nurse-patient integration, Endometriosis, Psychological status, Quality of life

## Abstract

**Background:**

To investigate the effect of applying doctor-nurse-patient integration management mode to patients with endometriosis (EMT).

**Methods:**

A total of 160 patients with EMT from the Department of Neurology of The Fifth Affiliated Hospital Sun Yat-Sen University from January 2017 to October 2017 were selected. They were divided into control group and experimental group according to the time sequence of hospitalization, with 80 patients in each group. The traditional nursing management mode was implemented in the control group, and doctor-nurse-patient integration mode was implemented in the experimental group. The psychological status, quality of life, and satisfaction of the 2 groups of patients were compared one year after surgery.

**Results:**

The anxiety and depression scores in the observation group were (41.89±7.50) and (42.40±7.40) points, respectively, and those in the control group were (57.55±9.68) and (55.00±9.35) points, respectively. The differences between the two groups were statistically significant (*t*=−11.44, −9.42, *P*<0.05). The improvement rate of quality of life (sleep, work and sexual life) in the observation group was 87.5%, which was higher than that in the control group (63.8%). The difference between the 2 groups was statistically significant (U=583, *P*<0.01). The satisfaction rate in the observation group was 90.00%, which was higher than that in the control group (78.75%) (U=592.00, *P*< 0.01).

**Conclusion:**

The doctor-nurse-patient integration management mode can effectively improve the negative psychological status and quality of life of patients with EMT and improve patient satisfaction, which is worth popularizing.

## Introduction

When endometrial tissue (gland and mesenchyme) appears outside the uterus, it is called endometriosis (EMT). Its main clinical manifestations are increased pelvic adhesion, pain and infertility. Although endometriosis is a benign disease, it has serious impacts on the patient’s physical and mental health and quality of life. At present, most treatment methods can only relieve symptoms, and there is still no effective cure method. Chronic pain brings negative psychological status to patients and reduces their quality of life (2).

In recent years, with the in-depth development of high-quality nursing service, more and more hospitals have explored the doctor-nurse-patient integration cooperation mode, and the doctor-nurse-patient integration management mode has been established. The overall services that integrate diagnosis, nursing and rehabilitation are provided to patients in the form of multidisciplinary integration and teamwork (3), which gives full play to the communication function of the nurses and has achieved good results.

In order to better study whether the doctor-nurse-patient integration cooperation mode was really applicable to patients with EMT, in this study, the doctor-nurse-patient integration mode was applied to patients with EMT.

## Materials and Methods

### Subjects

A total of 160 patients with EMT aged 20–54 yr old who were hospitalized in the Fifth Affiliated Hospital Sun Yat-Sen University undergoing surgery were enrolled in the study from January 2017 to October 2017. A total of 80 patients who went under the knife in our hospital from January to June 2016 (before implementation of doctor-nurse-patient integration) were selected as the control group. A total of 80 patients who volunteered to participate in the doctor-nurse-patient integration management from June to December 2016 were selected as the observation group. Inclusion criteria: ① patients with good communication skills, and ② patients who signed the informed consent. The traditional nursing mode was applied to the control group, and the doctor-nurse-patient integration service mode was applied to the observation group. There were no statistical differences in the general data between the 2 groups (*P*>0.05), and these data were comparable.

The study was approved by the Ethics Committee of The Fifth Affiliated Hospital Sun Yat-Sen University and written informed consents were signed by the patients and/or guardians.

### Methods

When the patients were enrolled in the groups, the basic conditions of the two groups of patients were investigated. Both groups of patients underwent surgery during the hospitalization, and the anxiety and depression scores and satisfaction surveys were performed for the two groups of patients on the day they were discharged from hospital. The routine nursing mode of gynecology was implemented in the control group. On the basis of routine nursing mode, the doctor-nurse-patient integration service mode was implemented in the observation group.

1) A service team consisting of nurses, gynecologists, psychological counselors, and volunteers was established. 2) The job responsibilities of team members were clarified. The gynecologists were responsible for the diagnosis and treatment of the patients, nurses for gynecological inpatient care, anxiety and depression assessment, health education and follow-up, psychological counselors for psychological counseling and intervention, and volunteers for life care (4). 3) The integrated service manuals were produced and the team members were trained, to enable them to follow the unified working process and standard. 4) The specific contents of doctor-nurse-patient integration service: a. During the hospitalization of the patients, the clinician and nurse communicated with the patients 3 to 4 times: The first communication was carried out after admission, the second communication was conducted when the diagnosis and treatment plan were adjusted or before the operation, the third communication was performed before discharge to allow the patients and their families participate in the decision-making and process of treatment. b. During the hospitalization, the patients were given routine nursing and examined and treated by gynecologists. At the same time, the nurses who had worked for more than 10 years first evaluated the patients one to one with the self-rating anxiety scale (SAS), the self-rating depression scale (SDS) and the self-restraint quality of life scale. The family members were allowed to participate in the evaluation at the same time for 20–40 min.

The evaluation was generally divided into three steps: First, the patients’ recent statuses were mastered. Then the nurses made judgments based on the patients’ self-evaluation results, and gave guidance to the patients on mental state and life care. Finally, the patients were added to the WeChat group composed of patients with endometriosis and the individual files were set up to carry out the whole-process case management. c. After the patients were discharged from the hospital after surgery, the nurses uploaded health information every week through WeChat platforms, online hospitals, and short message service reminders to disseminate knowledge on EMT and pain relief measures for dysmenorrhea, remind patients to adjust their sleep time, tell them dysmenorrhea pain relief methods and inform them about matters needing attention to sexual life, so as to establish a trust relationship among doctors, nurses and patients (5). d. One-to-one instructions were given to patients to listen to their needs and help to formulate personalized rehabilitation programs. At the same time, those with good recovery effect were introduced to communicate and share with these patients to help them reduce their anxiety and depression and establish their confidence in rehabilitation. When abnormality was found, early intervention was given. e. After discharge, the service was extended to families and communities. The hospital set up life nursing services together with community volunteers to solve the post-discharge life and psychological problems of the patients.

### Observation indicators

The improvement and satisfaction of the quality of life of the two groups of patients were observed. Emotional responses of patients before and after intervention were assessed using SAS and SDS, and the patient’s satisfaction with diagnosis and treatment was investigated one year after discharge. SAS and SDS contained 20 items, which were all scored using 4-grade criterion. According to the results of the Chinese norm, the SAS standard score of 50 points was the cut-off value of anxiety symptoms, and the SDS standard score of 53 points was the cut-off value of depressive symptoms (6). The higher the score, the more severe the symptoms. Satisfaction assessment was conducted in four specific aspects, namely, health nursing services, health education, and physical and mental experience during hospitalization. Satisfaction was divided into three levels: very satisfied, satisfied and dissatisfied. Satisfaction rate = Number of people who were very satisfied / Overall number of people *100%.

### Data processing

Statistical Product and Service Solutions (SPSS) 18.0 software (Chicago, IL, USA) was employed for data analysis, in which the measurement data that conformed to normal distribution were expressed as (x̄±s). The *t*-test was applied for the comparison between groups, and the *χ*^2^ test was adopted for enumeration data. Ranked data were compared using rank sum tests, and *P*<0.05 indicated a statistically significant difference.

## Results

### Comparisons of the anxiety and depression scores between the two groups of patients

There were no significant differences in the anxiety and depression scores between the two groups before intervention (*P*>0.05). The anxiety score in the observation group after the intervention was lower than that before intervention, and the difference was statistically significant (*P*=0.001). The depression score in the observation group after intervention was remarkably lower than that in the control group, with a statistically significant difference (*P*=0.001) (Table 1).

**Table 1:** Comparisons of anxiety and depression scores between the two groups of patients (points, x̄±s)

***Group***	***n***	***Anxiety score***			***P***	***Depression score***			***P***
		***Before intervention***	***After intervention***	***t***		***Before intervention***	***After intervention***	***t***	
Observation group	80	61.18±9.71	41.89 ± 7.50	−14.06	0.00	62.19±8.91	42.40±7.40	−15.23	0.00
Control group	80	62.19±8.91	57.55 ±9.68	−3.15	0.00	63.04±8.78	55.00±9.35	−5.59	0.00
*t* value		−0.69	−11.44			−0.61	−9.42		
*P*		0.49	0.00			0.54	0.00		

Note: The routine nursing mode of gynecology is implemented in the control group. On the basis of routine nursing mode, the doctor-nurse-patient integration service model is implemented in the observation group

### Comparison of improvement in quality of life between the two groups of patients

The improvement rate of quality of life in the observation group was higher than that in the control group, and the difference was statistically significant (*P*<0.01) (Table 2).

**Table 2:** Comparison of quality of life (work, sleep and sexual life) between the two groups of patients

***Group***	***n***	***Three of the quality of life improved (n)***	***Two of the quality of life improved (n)***	***One of the quality of life improved (n)***	***Improvement rate of three of quality of life %***
Observation group	80	70	6	4	87.5
Control group	80	51	12	17	63.8
*U*					583
*P*					0.00

Note: The routine nursing mode of gynecology is implemented in the control group. On the basis of routine nursing mode, the doctor-nurse-patient integration service model is implemented in the observation group.

### Comparison of nursing satisfaction between the two groups of patients

The very satisfied rate of the observation group was 90.00% (72/80), which was higher than that of the control group [78.75% (63/80)], and the difference was statistically significant (*P*=0.00) (Table 3).

**Table 3:** Comparison of nursing satisfaction between the two groups of patients

**Group**	**n**	**Very satisfied (n)**	**Satisfied (n)**	**Dissatisfied (n)**	**Very satisfied rate (%)**
Observation group	80	72	8	0	90.00
Control group	80	63	12	7	78.75
*U* value					592
*P*					0.001

Note: The routine nursing mode of gynecology is implemented in the control group. On the basis of routine nursing mode, the doctor-nurse-patient integration service mode is implemented in the observation group

## Discussion

### The doctor-nurse-patient integration service mode improves the quality of life of patients with EMT

In the aspect of the patient’s quality of life, on the basis of helping their patients to relieve symptoms, the medical staff also actively helps the patients to eliminate the psychological burden with a professional attitude (7). The medical staff enables the patient to fully understand their own condition, which is beneficial to the patient’s adjustment of their psychological state. Patient and meticulous explanation and guidance will have an important impact on the patient’s life. Concerned about this aspect, medical staff will spare time to communicate with patients as much as possible during the diagnosis and nursing. When diagnosis and nursing are performed, the assessment of the quality of life of patients with endometriosis should be valued. Regular life, adequate rest and proper diet can improve the body’s immune function. Not only do nurses provide routine cursing for patients, they also provide patients with the knowledge and information they need to improve their understanding of the disease, guide patients to develop healthy behaviors, and maintain optimal health condition.

### The doctor-nurse-patient integration service mode can reduce postoperative anxiety and depression in patients with endometriosis and improve patients’ nursing satisfaction

Postoperative mental health management and timely follow-up of patients with EMT can reduce postpartum depression and improve postoperative physical and mental health of them (8). The doctor-nurse-patient integration service mode provides patients with services integrating diagnosis, treatment, and rehabilitation in the form of multidisciplinary integration and team-work, which gives full play to the communication function of the nurses. In particular, nurses can develop professional skills, move the workplace forward, intervene in the post-discharge management stage, and provide individual counseling, health education, and post-discharge nursing for patients, thus dynamically observing the psychosomatic changes and social support of patients with EMT after discharge (9).

In the study, the anxiety and depression self-assessment scores of the patients in the observation group were significantly lower than those in the control group. This could be due to the facts that the patients had established a good relationship of trust with the medical staff, the bad emotions could be resolved in time, and the patients had adequate understandings and preparations for the disease. During the hospitalization, patients communicated with familiar doctors and nurses for many times to increase their psychological reliance and sense of security.

A series of continuous medical cursing services, such as follow-up after discharge, provided patients with a positive emotional experience in the rehabilitation of the disease and improved patient and family satisfaction. In the context of the new healthcare reform, the doctor-nurse-patient integration service mode is implemented, emphasizing the comprehensive and in-depth cooperation among doctors, nurses and patients, and giving full play to the subjective initiative of the three parties. This service mode not only benefits patients, but also allows nurses to go to the work-place outside the ward to obtain a sense of professional value and identity, which is worth recommending (10).

At present, the doctor-nurse-patient integration service in our hospital is still in the exploratory stage. How to establish the long-term management of multidisciplinary integration, use the information construction to improve the efficiency and mobilize the enthusiasm of the medical staff is the direction of our future research.

## Conclusion

The doctor-nurse-patient integration management mode can effectively improve the negative psychological status and quality of life of patients with EMT and improve patient satisfaction, which is worth popularizing.

## Ethical considerations

Ethical issues (Including plagiarism, informed consent, misconduct, data fabrication and/or falsification, double publication and/or submission, redundancy, etc.) have been completely observed by the authors.
